# Relationship of Metabolic Alterations and PD-L1 Expression in Cisplatin Resistant Lung Cancer

**DOI:** 10.4172/2168-9296.1000183

**Published:** 2017-04-28

**Authors:** M Wangpaichitr, H Kandemir, YY Li, C Wu, DJM Nguyen, LG Feun, MT Kuo, N Savaraj

**Affiliations:** 1 Miami VA Healthcare System, Research Service, Miami, Florida, USA; 2 Department of Surgery, Cardiothoracic Surgery, University of Miami, Miami, Florida, USA; 3 School of Medicine, Koc University, Istanbul, Turkey; 4 Department of Medicine, Hematology/Oncology, University of Miami, Miami, Florida, USA; 5 Department of Microbiology, University of Miami, Miami, Florida, USA; 6 Department of Translational Molecular Pathology, Texas MD Anderson, Houston, Texas, USA

**Keywords:** Lung cancer, PD-L1, EMT, Resistance, Cisplatin

## Abstract

Despite numerous reports on immune checkpoint inhibitor for the treatment of non-small cell lung cancer (NSCLC), the response rate remains low but durable. Thus cisplatin still plays a major role in the treatment of NSCLC. While there are many mechanisms involved in cisplatin resistance, alteration in metabolic phenotypes with elevated levels of reactive oxygen species (ROS) are found in several cisplatin resistant tumors. These resistant cells become more reliant on mitochondria oxidative metabolism instead of glucose. Consequently, high ROS and metabolic alteration contributed to epithelial-mesenchymal transition (EMT). Importantly, recent findings indicated that EMT has a crucial role in upregulating PD-L1 expression in cancer cells. Thus, it is very likely that cisplatin resistance will lead to high expression of PD-L1/PD-1 which makes them vulnerable to anti PD-1 or anti PD-L1 antibody treatment. An understanding of the interactions between cancer cells metabolic reprogramming and immune checkpoints is critical for combining metabolism targeted therapies with immunotherapies.

## Introduction

Treatment for early stage lung cancer is surgery but most patients already have locally advanced or metastatic disease at the time of diagnosis. Chemotherapy combined with radiation therapy or chemotherapy alone remains the primary modality of treatment for stage 3 and 4 disease. Targeted agents such as erlotinib or gefitinib (EGFR inhibitor) or crizotinib or ceritinib (ALK inhibitors) have shown activity in NSCLC (non-small cell lung cancer) which possess these putative types of mutation. However, both EGFR mutation and ALK mutation are rare (only 5-20%) and usually occur in women and non-smokers. Immunotherapy with checkpoint inhibitors has received much attention lately. They offer a longer duration of response; however, the response rate is still very low in lung cancer. In fact, a recent report on PD1 inhibitor (programmed death-1) did not show improved efficacy over standard chemotherapy as first line treatment in lung cancer and did not receive FDA approval as first line therapy for NSCLC. Another checkpoint inhibitor pembrolizumab has received FDA approval for first line treatment but only in tumors which express PD-L1 (program death receptor ligand-1). Therefore, platinum containing regimen remains the first line treatment in patient with NSCLC. Despite a 50% initial response rate to platinum-based chemotherapy, the majority of lung cancer patients develop resistance to treatment. Thus, cisplatin resistance remains the major impediment for the treatment of lung cancer.

Accumulating evidence suggests that tumor metabolism is in fact interconnected to drug resistance and it has proven to be one of the most important challenges in cancer treatment [1-3]. The observations of metabolic differences in cancer cells were first reported by Otto Warburg [4,5]. He showed that cancer cells prefer to utilize glucose even in the presence of oxygen; hence this led to the term “aerobic glycolysis”. This difference in energy metabolism between tumor and normal tissue has been utilized successfully in the development of a diagnostic imaging technique, fluoro-deoxy-glucose positron emission tomography (FDG-PET) for cancer detection. However, what is not known is why certain tumors are PET-negative (not taking up FDG), and why PET negativity does not always correlate with tumor response. Thus, it is conceivable that PET negative's tumors have undergone metabolic reprogramming after chemotherapy and are no longer addicted to glucose. To further support this notion, it has been shown that therapy-resistant tumors have altered metabolic phenotypes relative to treatment-naive tumors, with increased reliance on mitochondrial metabolism in the resistant cancers [6-9]. Increased mitochondrial metabolic activity can lead to high levels of reactive oxygen species (ROS) [10]. In fact, many have discovered that elevated reactive oxygen species (ROS) are found in cisplatin resistant (CR) cell lines including those derived from patients who failed cisplatin [11-14].

ROS, a harmful by-product of metabolism played an important role in signaling pathways. ROS is known to facilitate the activation of receptor tyrosine kinase signaling as well as PI3K/AKT which plays a vital role in cell growth/proliferation, survival, and motility [15,16]. Moreover, during the past decade, elevate ROS level in tumor cells have been implicated in epithelial-mesenchymal transition (EMT) [17-19]. Importantly, recent reports have shown that EMT played an essential role in upregulating PD-L1 (programmed death ligand-1) expression [20].

**Figure 1:**
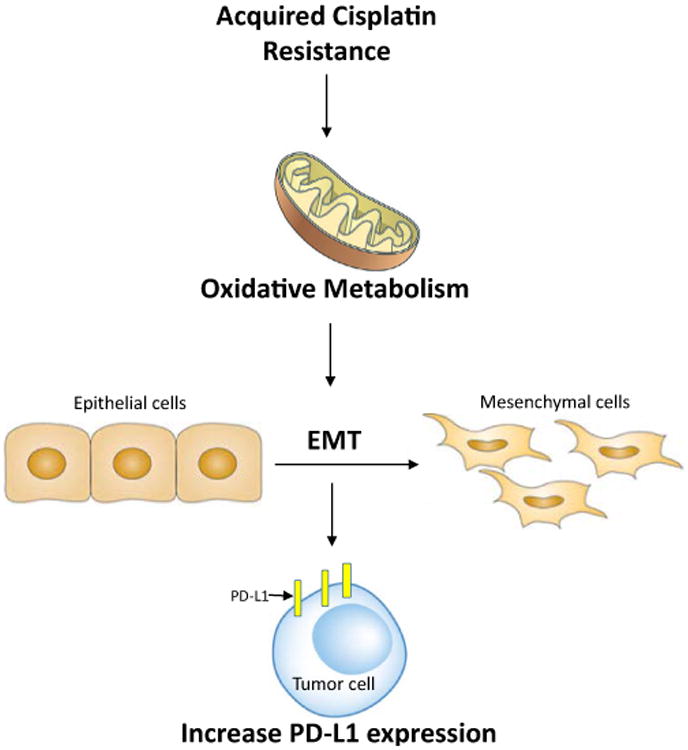
Acquired resistance to cisplatin results in accumulation of cellular ROS. Increased ROS levels involved in metabolic reprogramming by switching cisplatin resistant cells from glycolysis toward oxidative metabolism and triggers epithelial-mesenchymal transition (EMT). Furthermore, induction of EMT may lead to increase in PD-L1 expression in tumor cells.

In this review, we provide a possible link between metabolic alteration and PD-L1 expression in cisplatin resistant lung cancer (Figure 1). Understanding these complex interrelationships will provide a new approach in overcoming the cisplatin resistance in lung cancer.

### Cancer cells and their carbon sources

It is known that most if not all tumors utilize glycolysis instead of oxidative phosphorylation (OXPHOS) (4, 5). This is due to up-regulation of glycolytic enzymes and glucose transporters [21,22]. In fact, increased glucose uptake is one of the hallmarks for malignant transformation [23,24]. Recently, it has been shown that up-regulation of pyruvate kinase-M2 (PKM2), an enzyme in the glycolytic pathway which converts phosphoenolpyruvate (PEP) to pyruvate, could be an answer for the aerobic glycolysis observed in Warburg's theory. PKM2 is a key protein in directing tumor cells toward glycolysis [25]. PKM2 increases the DNA binding of HIF1α. Consequently, increases in HIF1α target gene expression. Cells expressing high levels of PKM2 are known to consume less oxygen and produce more lactate [25]. On the other hand, reduced PKM2 activity allows accumulation of glucose-6-phosphate and thus shifts glucose flux toward the pentose phosphate pathway (PPP) to generate reduced NADPH. Consistent with this notion, acute increases in intracellular concentrations of ROS caused inhibition of the glycolytic enzyme pyruvate kinase M2 (PKM2) through oxidation of Cys^358^ in lung cancer cells [26].

Although increased glucose metabolism in cancer cell has been recognized as the main carbon skeleton source of energy, we and others [27] have shown that cisplatin resistant (CR) cells are no longer addicted to the glycolytic pathway [11,28]. CR cells use other carbon sources to replenish TCA cycle intermediates (anaplerosis) for their energy demand and biosynthesis. In this regard, reports have shown that certain tumor types are highly addicted to glutamine [29,30]. The cellular demand in these tumors outstrips its glutamine supply; hence glutamine becomes the conditionally essential amino acid. Moreover, studies have shown that reduction in lactate dehydrogenase A (LDHA) expression in cancer cells either by genetic knock down (shRNA) or inhibitor (FX11) resulted in the shift to oxidative phosphorylation (OXPHOS) and increased intracellular ROS [31,32]. Therefore, there is no doubt that alteration in metabolism has gained its status as a core hallmark of cancer.

### Redox and oxidative metabolism (OXMET)

A number of investigators have shown that cisplatin can inhibit thioredoxin reductase (TrxR) which leads to increased ROS. As a result, DNA damage occurs which can lead to cell death [33,34]. In order to adapt and survive at higher ROS levels and to evade cell death caused by cisplatin, CR cells use less thioredoxin-1 (TRX1) and employ other antioxidant systems to compensate for the lack of TRX1 [35,37]. In fact, many reports showed that CR cells have higher level of glutathione (GSH) proteins [12,38,39]. Lower intracellular TRX1 also was not due to the protein degradation caused by cathepsin-D [40], but as a consequence of increased TRX1 secretion. TRX1 is secreted via special secretory pathway called “leaderless pathway”. This pathway is known to secrete low molecular weight proteins which lack signal peptide [41-43]. The mechanism of how this pathway functions remains poorly understood. Nevertheless, increased TRX1 secretion usually occurs when cells are under stress [44-46] and is found in patients who received cisplatin treatment [47,48]. In fact, many investigators have reported that higher serum TRX1 resulted in bad prognosis and drug resistance [49,51]. Decreased intracellular TRX1 has also been shown to reprogram lung cancer cells to become more reliant on oxidative metabolism (OXMET) [11] and overexpression of Txnip, an inhibitor of TRX1, can lead to adipogensis [52]. Thus, these findings could have future implication for drug development to selectively kill CR cells that have high ROS and low TRX1 levels.

### ROS triggers epithelial-mesenchymal transition (EMT) in cancer cells

One of the first studies that established a direct connection between ROS and EMT was reported in the cross-talk signaling between ROS and TGF-β [53]. TGF-β stimulated ROS production was responsible for E-cadherin repression [53]. EMT related molecular events can also be stimulated by H_2_O_2_ treatment. It was noteworthy that the crucial event in EMT is represented by the disassembly of the epithelial structure and thus, E-cadherin down regulation was the most relevant step [19,54]. Several transcription factors contributed to this event for allowing E-cadherin repression. One of the important factors was the two zinc fingers E-box binding homeobox transcription factor ZEB1 and ZEB2 [55]. While ZEB1 is known to repress T lymphocytes IL2 gene expression, ZEB2 (previously known as SMADIP1, SIP1) can activate TGFβ. These repressors collaborate with histone deacetylases and histone demethylases, ensuring the maintenance of the silenced state of the E-cadherin gene [56].

### The relationship between EMT and PD-L1 regulations

EMT is known as a driving force for metastasis and drug resistance. The presence of EMT signifies poor prognosis in many tumors including NSCLC [57]. Another factor which dictates tumor cells behavior is the immune cells in the tumor microenvironment. Studies have been carried out in lung adenocarcinoma which showed an increase in inflammatory signal cytokines such as IFN gamma and immune checkpoints markers including PD-L1/2, PD1, TIM3, B7H3, BTLA, LAG3 and CTLA4 [57]. Correlation between EMT and PD-L1 expression [58-60] is illustrated in Table 1. Furthermore, induction of EMT increased immunosuppressive cytokines and increased immunosuppressive CD8+ tumor infiltrating lymphocytes in preclinical models of lung, melanoma, pancreatic cancer, and breast cancer [61-63]. Importantly, microRNA200 and ZEB1 axis, which is known to control cancer cell migration/invasion and EMT, can also regulate PD-L1 expression. Decrease in PD-L1 expressions was reported as a consequence of ectopic microRNA 200 expression or ZEB1 knockdown models [20]. In fact, low microRNA200 with high ZEB1 and PD-L1 expressions in mesenchymal tumors created a microenvironment of decreased CD8+ T-cells populations [20].

PD-L1, a ligand of PD1 is an immune regulatory protein deriving from B7 family of T-cell co-regulatory molecules [64]. Their interaction prevents T-cell activation and proliferation including cell apoptosis and creates cancer resistance. So far, PD-L1 was found in many solid neoplasms such as cancer of the breast, colon, esophagus, stomach, ovaries, pancreas and lung [64]. As a prognostic marker, PD-L1 expression is a poor prognostic factor for gastric cancer, liver cancer, esophageal cancer, ovarian cancer, bladder cancer, but served as a better prognostic factor for breast and merkel cell carcinoma [65], while remains controversial in melanoma and lung cancer. Many reports suggest that EGFR and KRAS mutations contribute to increase in PD-L1 expression; however, the molecular mechanism behind this important biochemical event remains to be elucidated [66,67].

### Drug resistance and PD-L1 expression

Recent studies have shown that treatment with cisplatin, carboplatin, paclitaxel, and 5-FU contribute to acquired PD-L1 expression in many solid tumors including small cell lung cancer (SCLC) and NSCLC [64,68,69]. Knocking-down PD-L1 was able to overcome cisplatin resistance. Further investigations also supported PD-L1 as the main resistance mechanism against cisplatin in SCLC via the over expression of DNMT1 or KIT. Down-regulation of these two proteins showed less PD-L1 expression and could overcome cisplatin resistance in H60 and H82 cell lines [69]. Thus, targeting the cellular PD-L1 may hyper-sensitize aggressive lung cancer to standard chemotherapy.

## Concluding Remarks

**Figure 2:**
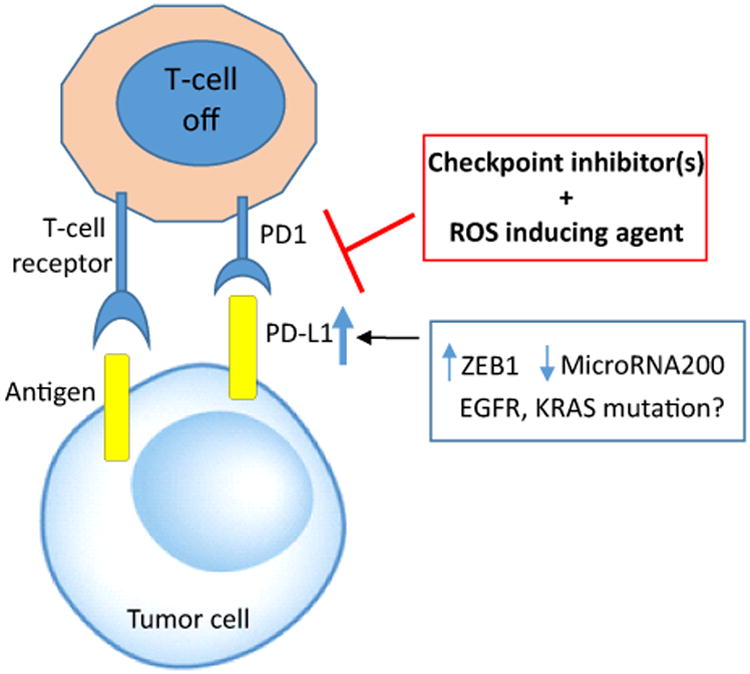
Cancers cells adapt and exploit immune system to evade immune surveillance by activating PD-L1/PD1 axis. ZEB1 and microRNA200 can regulate this axis. KRAS or EGFR mutation may also influence PD-L 1 expression. Blocking PD1 and PD-L1 interaction with checkpoint inhibitor(s) in combination with ROS inducing agent may lead to new approaches to overcome cisplatin resistant lung cancer.

Immunotherapy with checkpoint inhibitors has received much attention lately. This type of therapy offers a longer duration of response; however, the response rate is still low in lung cancer. In fact, a recent report on immunotherapy did not show improved efficacy over standard chemotherapy and failed as first line treatment in lung cancer. Therefore, the majority of lung cancer patients still require the traditional chemotherapeutic agents such as cisplatin or carboplatin to control their disease. We have found that the major biochemical alterations in cisplatin resistance are increasing ROS and metabolic reprograming which can be used to kill cisplatin resistant cells. Furthermore, the tumor microenvironment may also be modified in these resistant tumors by multiple factors including immune cells such as tumor-infiltrating lymphocyte. These resistant cells undergo epithelial-mesenchymal transition to enable invasion/metastasis as well as escape immune surveillance by expressing PD-L1/PD1. Combination of ROS inducing agent with immunomodulation approach may ultimately lead to cisplatin resistant cell death (Figure 2).

## Figures and Tables

**Table 1:** Correlations between PD-L1 expression and EMT [58-60].

Head and Neck Cancer N=50	PD-L1 positive 64%	Associate with EMT (Low E-cadherin expressions p=0.010	Ock et al. 2016
Lung Adenocarcinoma N=220	PD-L1 positive 42%	Associate with EMT (Low E-cadherin expressions) p<0.001	Shimoji et al. 2016
Lung Adenocarcinoma N=477	PD-L1 positive 60%	Associate with EMT (High vimentin expressions) p<0.01	Kim et al. 2016
